# Supplementary material for: IRES-dependent ribosome repositioning directs translation of a +1 overlapping ORF that enhances viral infection
Source: Nucleic Acids Res. 2018 Nov 10;46(22):11952–67. doi: 10.1093/nar/gky1121 (PMC6294563; doi:10.1093/nar/gky1121)

Kerr_Figure S1

Dicistrovirus	IGR IRES Type	Potential +1 basepair	Predicted +1 ORFx Length (aa)	First +1 frame amino acid
Solenopsis invicta virus-1 (SINV-1)	II	C-G	125	L
Israeli acute paralysis virus (IAPV)	II	U-G	94	A
Kashmir bee virus (KBV)	II	C-G	93	L
Acute bee paralysis virus (ABPV)	II	U-G	92	P
Cricket paralysis virus (CrPV)	I	U-G	53	L
Drosophila C virus (DCV)	I	U-G	51	L
Plautia stali intestine virus (PSIV)	I	U C (?)	43	K
Big Sioux River virus (BSRV)	I	U-G	40	L
Black queen cell virus (BQCV)	I	U-G	26	L
Homalodisca coagulata virus- 1	I	U-G	17	Q
Taura Syndrome virus (TSV)	II	U-G	16	L
Himetobi P virus (HiPV)	I	U-G	14	Q
Bat guano dicistrovirus	I	U-G	14	Q
Mud crab dicistrovirus (MCDV)	II	U-G	9	L
Aphid lethal paralysis virus (ALPV)	I	U-G	7	L
ALPV like Brookings virus	I	U-G	7	Q
Rhopalosiphum padi virus (RhPV)	I	U-G	2	Q
Triatoma virus (TrV)	II	C-G	1	L

Kerr_Figure S2

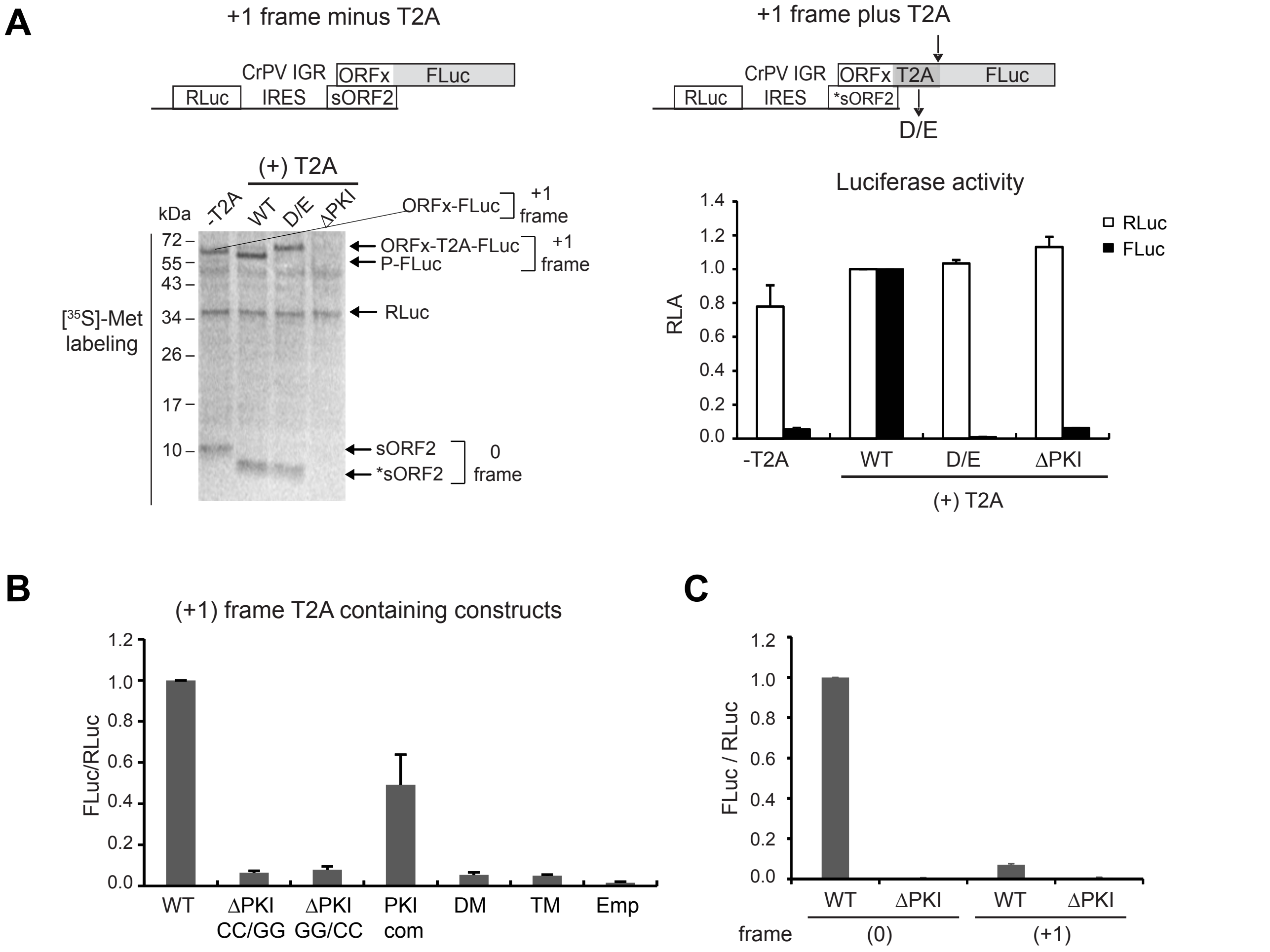

Kerr_Supplemental Figure 3

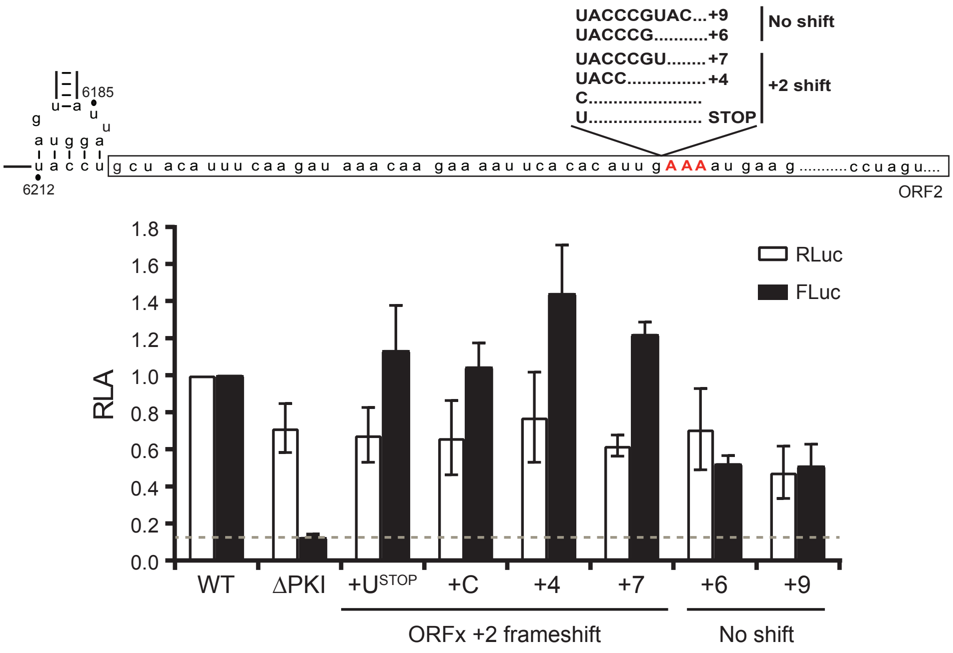

Kerr_Supplemental Figure 4

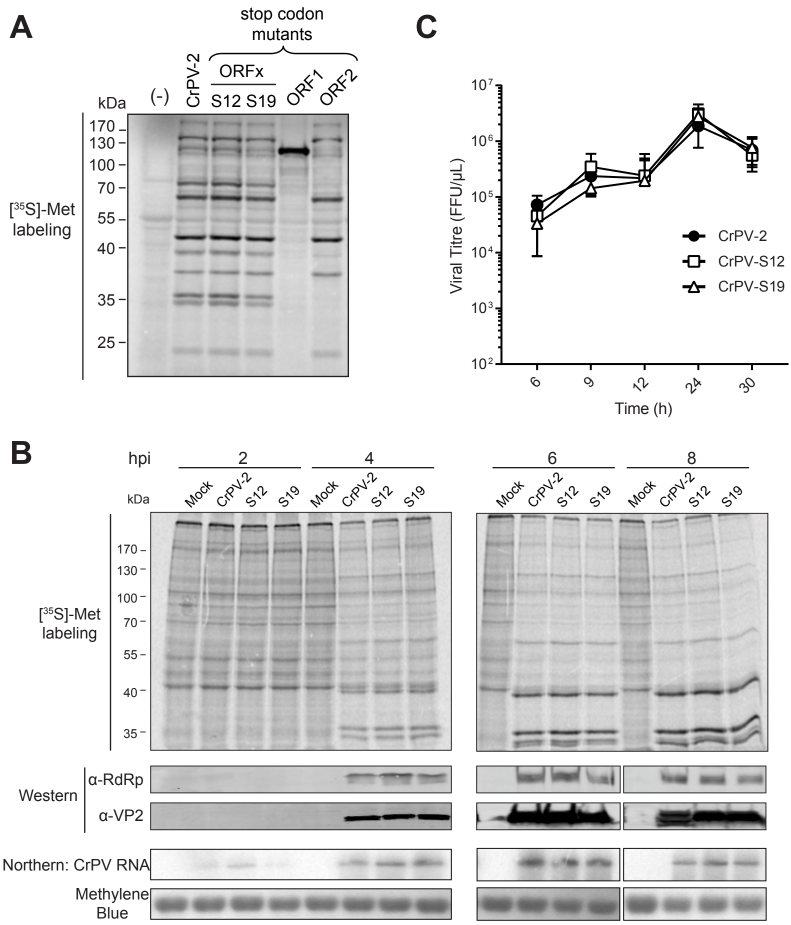

Kerr_Supp Figure 5

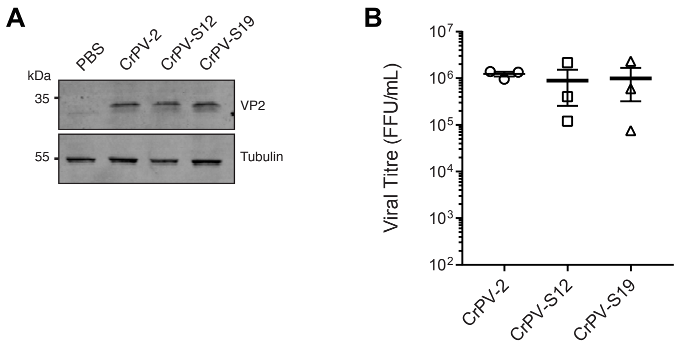

Kerr_Supp Fig 6

A

CrPV ORFx: KMKIKDLCQNRKKLYILLAKELPLVLLRSLISLIFQLIIWT

IAPV ORFx: AIHNKKAILPTYTIRNSLRPLVKTRLRPKKSQPFMMWKLQIGSIPPWLRILHRLGTWMIRTVLFSFYSAPFSLTTLRSLLEQRPMQTNPLADMC

B

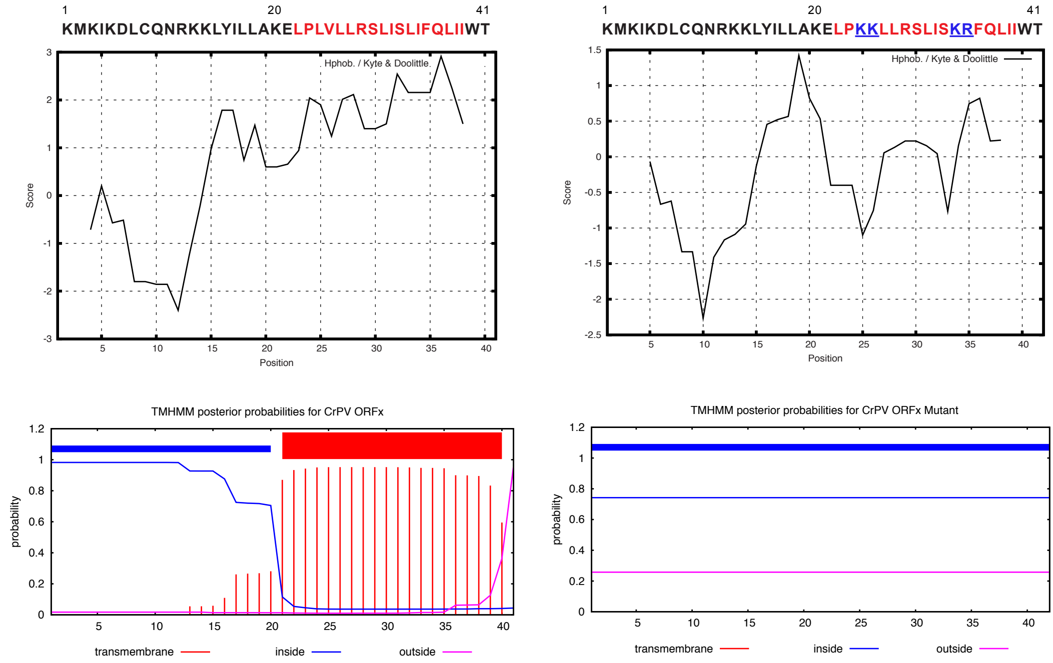

C

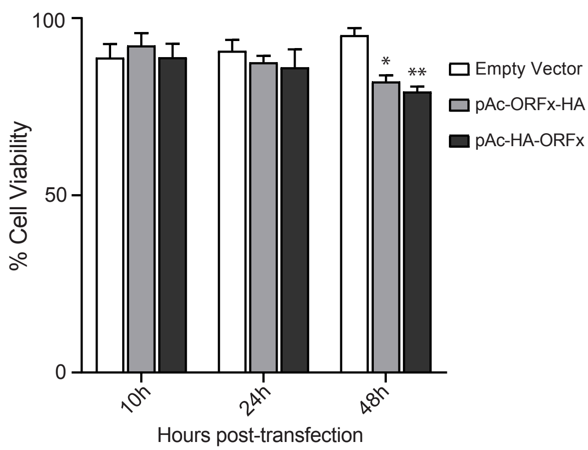

D

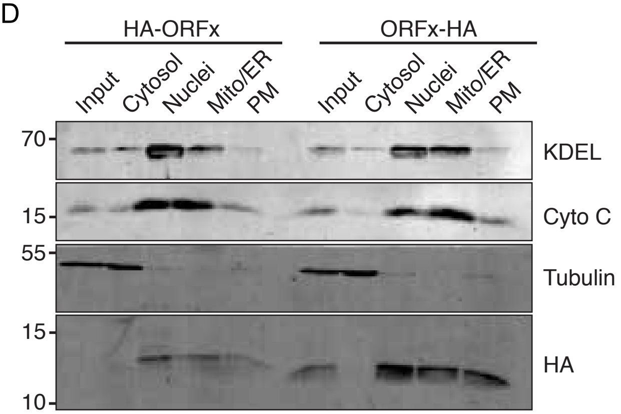

Kerr_Supp Figure 7

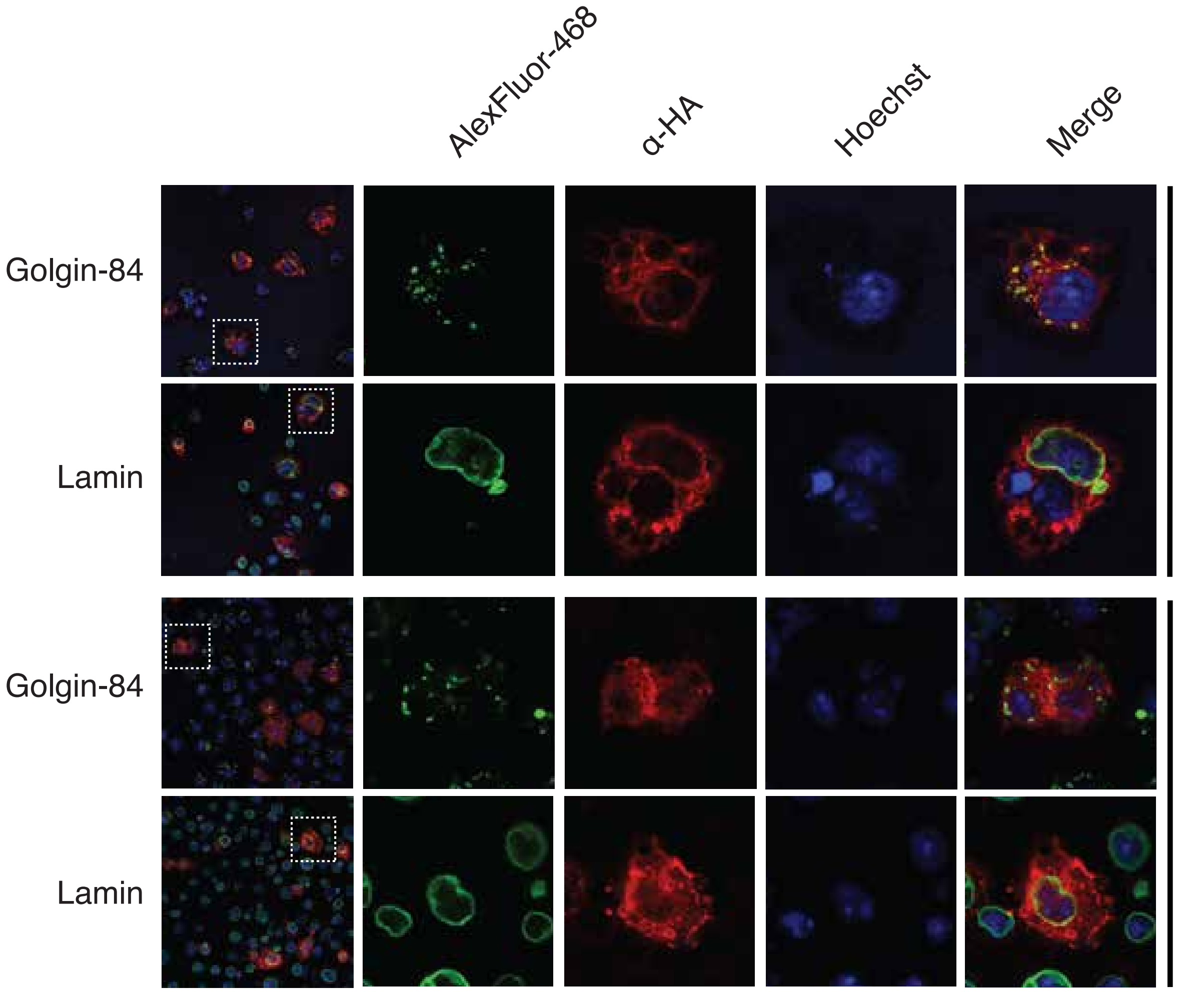

Kerr_Figure S8

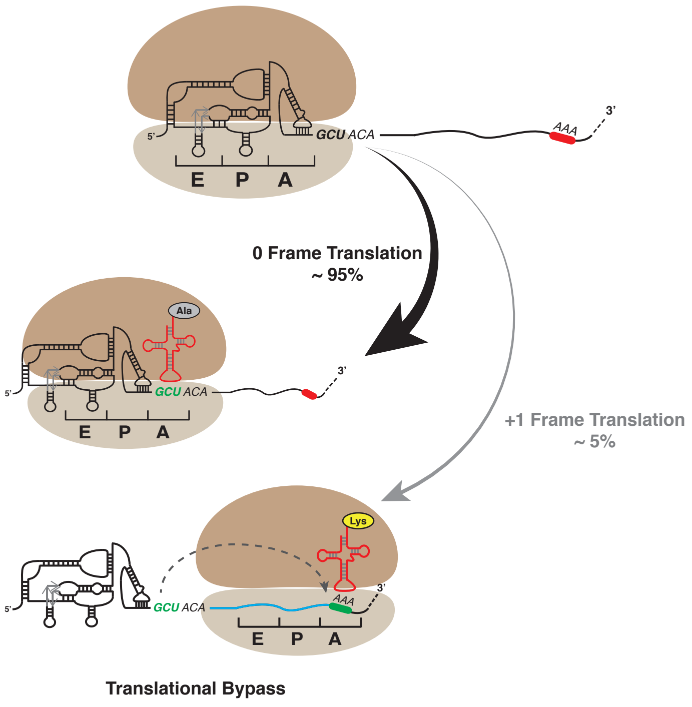

Supplement: Supplementary Data [file gky1121_supplemental_files.zip › KERR SUPPLEMENTAL FIGURES OCT 10.pdf]
