# Supplementary material for: IRES-dependent ribosome repositioning directs translation of a +1 overlapping ORF that enhances viral infection
Source: Nucleic Acids Res. 2018 Nov 10;46(22):11952–67. doi: 10.1093/nar/gky1121 (PMC6294563; doi:10.1093/nar/gky1121)
Supplement: Supplementary Data [file gky1121_supplemental_files.zip › Kerr_ORFx_July 31 2018 supplemental .docx]

**SUPPLEMENTAL INFORMATION**

**SUPPLEMENTAL FIGURE LEGENDS**

**Fig. S1. *In silico* analysis for potential +1-frame translation mediated by IGR IRESs in Dicistroviruses.** List of type I and type II IGR IRESs based on their secondary structures. Potential +1-frame base pair immediately downstream of their IGR IRES PKI region as in IAPV IGR IRES, together with the predicted length of ORFxs and the first amino acids, are shown.

**Fig. S2**. **Construction of a** **T2A construct +1-frame reporter construct that recover FLuc enzyme activity. A)** The schemes of bicistronic constructs in which firefly luciferase gene (FLuc) is in frame with the CrPV ORFx are shown. The T2A sequence (dark grey) is inserted between the CrPV ORFx and the FLuc gene. The arrow indicates the ‘self-cleavage’ or ‘stop-go’ site. A mutation within the T2A peptide (D to E) inactivates T2A ‘self-cleavage’ activity. T2A-minus (-T2A) and T2A-containing ((+)T2A) +1-frame bicistronic constructs were incubated in Sf21 extracts for 2 h in the presence of [^35^S] methionine and cysteine. The left panel is a representative SDS-PAGE monitored by autoradiography, whereas the right panel is the quantitation of relative luciferase activity (RLA) measured by Dual-Luciferase reporter assay (Promega). RLA are normalized to that observed with the +1-frame T2A-containing reporter construct. Shown are averages from at least three independent biological experiments (± SD). **B)** The quantitation of luciferase activities of IGR IRES mutants compared to WT T2A containing +1-frame constructs are shown. Displayed are averages from at least three independent biological experiments (± SD). **C)** The comparison between +1-frame and 0-frame T2A containing constructs. The ratio of +1/0-frame translation *in vitro* is 7.1 ± 0.5 %, consistent with that monitored by autoradiography using T2A-minus constructs (Fig. 1B). Note that the FLuc activity levels from both the 0-frame ΔPKI mutant and the +1-frame ΔPKI mutant indicates to some extend IGR IRES independent translation. Shown are averages from at least three independent biological experiments (± SD).

**Fig. S3. CrPV IRES-mediated +2 frame translation.** Mutations and insertions that shift the ORFx into the +2 frame are shown in the schematic above. Translation assays were monitored and normalized to that of the wild-type CrPV IRES. Shown are averages from at least three independent biological experiments (± SD).

**Fig. S4. Role of CrPV ORFx in CrPV-infected S2 cells. A) CrPV ORFx has no effect on viral protein synthesis *in vitro*.** The *in vitro* transcribed RNA from CrPV-2 or the cognate mutants was incubated in Sf21 cell extract at 30°C for 2 hours in the present of [^35^S]-methionine/cysteine. Translation of viral proteins were monitored by autoradiography using 12% SDS PAGE. A representative gel of at least three independent biological experiments is shown. Note that CrPV ORFx is predicted to be ~4.9 kDa and is undetectable in this analysis. **B)** **CrPV ORFx has no effect on viral protein synthesis and RNA replication in S2 cells.** The *in vitro* transcribed RNA of CrPV-2, S12, or S19 mutants was transfected into S2 cells. Virus was harvested and used to infect naïve S2 cells at MOI 10. Cells were metabolically labeled with [^35^S]-methionine/cysteine for 30 min at 2, 4, 6 and 8 h.p.i. Lysates were collected and resolved on a 12% SDS PAGE. Western blot analysis against the CrPV RdRp and VP2 and Northern blot analysis of viral RNA are shown. Shown is a representative gel from three independent biological experiments. **C) CrPV ORFx has no effect on viral replication in S2 cells.** S2 cells were infected with CrPV-2, CrPV-S12, or CrPV-S19 viruses at MOI 1.0. Viral titres were measured at 6, 9, 12, 24, and 30 h.p.i for MOI 1. Shown are average from at least three independent biological experiments (±SD).

**Fig. S5.** **Role of ORFx in CrPV-infected adult fruit flies.** **A)** Flies were harvested at 5 days post injection (dpi) and homogenized in PBS. Viral titres were determined as described in materials and methods. Error bars represent the standard error of the mean. **B)** Western blot against CrPV VP2 on lysates from flies 5 dpi. Shown is a representative gel from at least three independent biological experiments.

**Fig. S6. Cytotoxicity of CrPV ORFx expression in Drosophila S2 cells.** **A) Comparison of CrPV and IAPV ORFx amino acid sequence. B) The transmembrane helix property of CrPV ORFx.** The predicted CrPV ORFx sequence stemming from the 13^th^ codon was analyzed for transmembrane helices using ProtScale and HMMTOP. The mutated form of ORFx is shown on the right. Shown above are hydropathy plot outputs from ProtScale and below are HMMTOP predictions. The red amino acid region indicates the putative transmembrane helix predicted by HMMTOP. **C) Ectopic expression of CrPV ORFx causes slight cytotoxicity in S2 cells.** S2 cells (1.5 x 10^6^) were transfected with expression constructs containing N- or C-terminally HA-tagged ORFx or empty vector and monitored over the course of 48 hours for cell death via trypan blue staining. * = p-value < 0.05. **D) CrPV ORFx associates with membrane fractions.** S2 cells were transfected with expression constructs encoding HA-tagged ORFx at the N- or C-terminus for 48 hours. Cells were then lysed and fractionated by differential centrifugation (see material and methods). Fractions were then analyzed by Western blot to detect the presence of CrPV ORFx or cellular markers. Shown is a representative gel from two independent biological experiments.

**Fig. S7. Subcellular localization of CrPV ORFx.** S2 cells were transfected with a construct expressing C-terminally HA-tagged ORFx or a mutant version of ORFx in the transmembrane region and incubated for 48 h. Following incubation, cells were fixed, permeabilized and co-stained with HA antibody and antibodies against nuclear lamina (Lamin) and the Golgi apparatus. (Shown are representative Z-stack micrographs from three independent experiments. Scale bars represent 15 µm.

**Fig. S8. Model of CrPV IGR IRES mediated +1-frame ORFx translation.** The majority of ribosomes bound to the IGR IRES initiates translation in the 0-frame from the GCU start codon. A subset of ribosomes bound to the CrPV IGR IRES (~5%) initiates translation in the 0 frame and then repositions downstream at the +1 frame 13th codon (AAA) to translate ORFx. The model proposes the AAA codon is vacant to allow delivery of the Lys-tRNA.
